# Tracking the recent dynamics of Mt. Vesuvius from joint investigations of ground deformation, seismicity and geofluid circulation

**DOI:** 10.1038/s41598-020-79636-w

**Published:** 2021-01-13

**Authors:** Ciro Ricco, Simona Petrosino, Ida Aquino, Paola Cusano, Paolo Madonia

**Affiliations:** 1 Istituto Nazionale di Geofisica e Vulcanologia, Sezione di Napoli - Osservatorio Vesuviano, via Diocleziano 328, 80124 Naples, Italy; 2 Istituto Nazionale di Geofisica e Vulcanologia, Sezione di Roma 2, via di Vigna Murata 605, 00143 Rome, Italy

**Keywords:** Solid Earth sciences, Geochemistry, Geophysics, Hydrogeology, Seismology, Volcanology

## Abstract

We reconstruct the composite dynamics of Mt. Vesuvius volcano in the period 2012–2019 from the study of ground deformation, seismicity, and geofluid (groundwater and fumarolic fluids) circulation and recognize complex spatio-temporal variations in these observables at medium (years) and short (months) time-scales. We interpret the observed patterns as the combined effect of structural changes affecting the volcanic edifice and variations of the dynamics of the hydrothermal system. In particular, we identify a change in the activity state of Mt. Vesuvius. After the activity reached minimum levels in 2014, the centroid of the surface manifestations migrated towards the SE. Episodic variations of co-seismic and aseismic deformation and fluid release, if analysed separately, would likely have been interpreted as pseudo-random oscillations of the background geophysical and geochemical signals. When organised in a comprehensive, multiparametric fashion, they shed light on the evolution of the volcano in 4D (x,y,z, time) space. These inferences play a crucial role in the formulation of civil protection scenarios for Mt. Vesuvius, a high risk, densely urbanized volcanic area which has never experienced unrest episodes in the modern era of instrumental volcanology.

## Introduction

One of the most effective approaches to investigate the dynamics of volcanoes is joint analysis of geophysical and geochemical data^1–3^. Detecting anomalous variations from background oscillations helps to identify significant changes of the volcanic activity state^4–6^. Prompt detection of such variations is particularly relevant for densely populated volcanic areas, among which Mt. Vesuvius represents one of the most potentially dangerous situations in the world.

**Figure 1:**
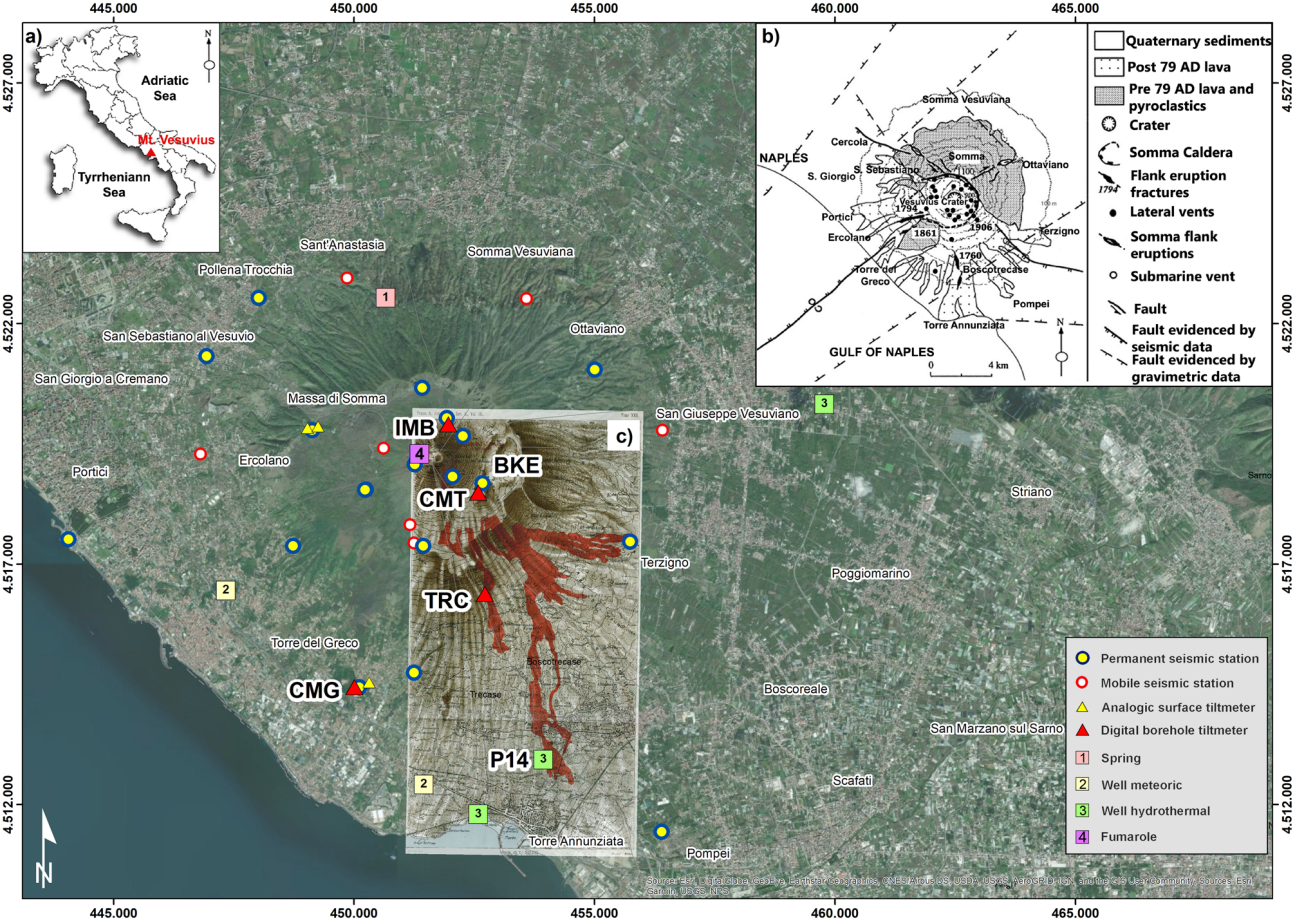
Seismic, tiltmeter and geochemical monitoring networks at Mt. Vesuvius used for the present analysis (base map of ArcGIS Desktop, ESRI, https://desktop.arcgis.com/en/arcmap/); (**a**) location of the study area; (**b**) structural sketch map of the volcano (modified after Bianco et al.^7^); (**c**) historical map of eruptive vents and the lava flow formed during the 1906 eruption (courtesy of Dr. Eliana Bellucci Sessa).

The Somma-Vesuvius complex (1281 m asl high and 10 km wide) is a stratovolcano located in the Campanian Plain at the intersection of two regional tectonic fault systems (NW–SE and NE–SW), with eruptive fractures trending E–W and N–S (Fig. 1). A wide gravimetric anomaly is located beneath the volcanic edifice, caused by the subsidence of the underlying (2 km depth) carbonate basement^8,9^. Volcanic activity has been characterized by alternating explosive and effusive phases, separated by periods of quiescence^10,11^. The last activity period started after the 1631 A.D. sub-Plinian eruption and terminated in 1944, when lava emitted from the Gran Cono crater ran northward along the valley separating Mt. Vesuvius from Mt. Somma (Valle dell’Inferno) and flowed down to the city of San Sebastiano. During this eruptive period there were many eruptions of different energy, including the one of 1906 (VEI = 4), called "the great eruption", that was the most violent of the twentieth century. During this eruption, fractures opened on the SE side of the Gran Cono, creating an area of instability with formation of lateral vents at different elevations (1200, 800 and 600 m asl) (Fig. 1). The volcano ejected ballistic products to distances exceeding 4 km in the ENE direction, with huge ash emissions in the SE direction^12,13^.

Since then Mt. Vesuvius has been in a state of weak activity, characterized by low seismicity, general subsidence, with maximum values on the Gran Cono^14^, and release of magmatic-hydrothermal fluids in groundwater through diffuse CO_2_ degassing and low temperature (T < 100 °C) fumarolic emissions in the crater area and undersea^15–17^.

The seismicity of Mt. Vesuvius consists mainly of volcano-tectonic (VT) earthquakes caused by shear failure mechanisms^18,19^. In recent decades the earthquakes originate in two distinct seismogenic volumes: the first is located within the volcanic edifice, above sea level; the second is at depths between 1–6 km bsl. In both cases, the centroid of the hypocenter locations is less than 2 km from the crater axis^18,19^. Seismicity within the shallow volume is usually characterized by low magnitude (duration magnitude, Md < 2.8) and quite stationary strain release. Higher magnitude shallow seismicity has been related to fluid-driven rock fracturing triggered by pressure variation in the shallow aquifer^1,18^. The deeper earthquakes, which occur preferentially in high-energy seismic swarms, have higher magnitudes (up to 3.6 for the earthquake recorded on October 9th 1999), and are mainly related to the interaction of the regional and local stress field, as well as fluid circulation in the hydrothermal system^1,16,18,20^. Between the 1999–2000 and the end of 2011, seismicity consisted mainly of low-magnitude earthquakes within the shallow volume^18^. Analysis has also revealed the occurrence of atypical earthquakes with a lower frequency content than that of the VTs. On July 20th 2003 a Long-Period (LP) earthquake was detected for the first time; this event was located at a depth of about 4 km bs1^21^ and associated with perturbations in the deep hydrothermal system^18,20,22^. Other unusual events were observed in the years 2012–2016 and classified as low frequency deep earthquakes, likely generated by a slow brittle rupture mechanism^23^. Recently, Petrosino and Cusano^24^ analysed in detail the seismicity from 2003 to 2018, recognizing 48 atypical events. These earthquakes were interpreted as the response of a NaCl brine reservoir, located between 2 and 5 km bsl^25^, to episodic pressurization/depressurization. The authors separated Low-Frequency (LF) and LP events based on the possible triggering mechanisms: brittle slow failure of dry rocks *versus* resonance of pre-existing fluid-filled cracks.

The kinematics inferred from more than twenty years of tiltmeter signals through 2011 showed a complex and site-dependent ground tilt pattern, constrained by the morphology of the volcanic edifice and its structural outlines^26^. Nevertheless, over long time-scales, all tilt vectors have a common orientation, supporting the hypothesis of subsidence of the southern part of the volcanic edifice. This behavior is consistent with the spreading inferred from geological, structural, geophysical and geodetic data (optical levelling, DInSAR)^27^. Despite the typical subsidence pattern, there have been periods where the preferential directions have changed more or less gradually. In fact, the background tilt pattern showed interruptions in both trend and amplitude during two phases of strong seismic activity that affected Vesuvius between 1995 and 1996, and the end of 1999. These seismically active periods were characterized by an average energy release rate of at least one order of magnitude higher than the previous and subsequent periods^23^. Moreover, from the end of 2000 to early 2001 there was a strong reversal of the tilt directions, at the beginning of a period of significant reduction in the local seismic energy release^23^. These observations indicate a close link between ground tilting and seismic activity.

According to previous studies, the groundwater circulation system of Mt. Vesuvius is rooted in a basal carbonate aquifer receiving recharge from the Apennines and subdivided in two parts: the southern and western sectors, where contributions of deep fluids are more evident, and the eastern and northern sectors, dominated by shallow meteoric fluids (see Federico et al.^22^ and references therein). Other surface manifestations of hydrothermal circulation are found in a low temperature (t < 100 °C) fumarolic field covering the rim and the inner flanks of the Gran Cono crater, associated with diffuse soil CO_2_ emissions^28^.

Temperature of groundwater and of the fumarolic field have been monitored by discrete surveys since 1998 and by continuous monitoring stations (hourly acquisition) since 2005^1,18,22,29^. The general framework depicted by the converging results from these studies is that the hydrothermal system of Mt. Vesuvius has been experiencing a phase of decreasing activity interrupted by episodes of enhanced release of deep fluids from 1998–2010. The main episode of deep fluid release occurred in 1999 and affected the hydrothermal circulation system of the volcano^22,29^, while a later event (2005) was evidenced only by changes at the summit fumarolic field of the Gran Cono^1,18^. In both cases the degassing episodes were apparently driven by permeability variations accompanying seismogenic processes that are interpreted as mainly tectonic.

The recent activity state of Mt. Vesuvius has never been interrupted by episodes significant enough to be classified as unrest, even at a very early stage, making difficult to define scenarios upon which operative civil protection procedures could be based (i.e. transition between different alert conditions). This is a very serious issue for the monitoring of a volcano like Mt. Vesuvius, enveloped in the vast conurbation of Naples, which has been one of the most densely inhabited areas of the world for millenia.

With the aim of better defining the space/time evolution patterns of volcanic activity at Mt. Vesuvius, we present and discuss seismicity, ground tilt, and geochemical data related to magmatic/hydrothermal fluid circulation in groundwater and the fumarolic fields. Among the geochemical parameters, we focused on temperature and bicarbonate contents of groundwater and fumarole temperature, since these are good proxies for flux variations of the two main gaseous magmatic species released at Mt. Vesuvius: water and carbon dioxide. The studied period goes from 2012, when a full suite of information became available, to 2019.

## Results

### Seismicity

**Figure 2:**
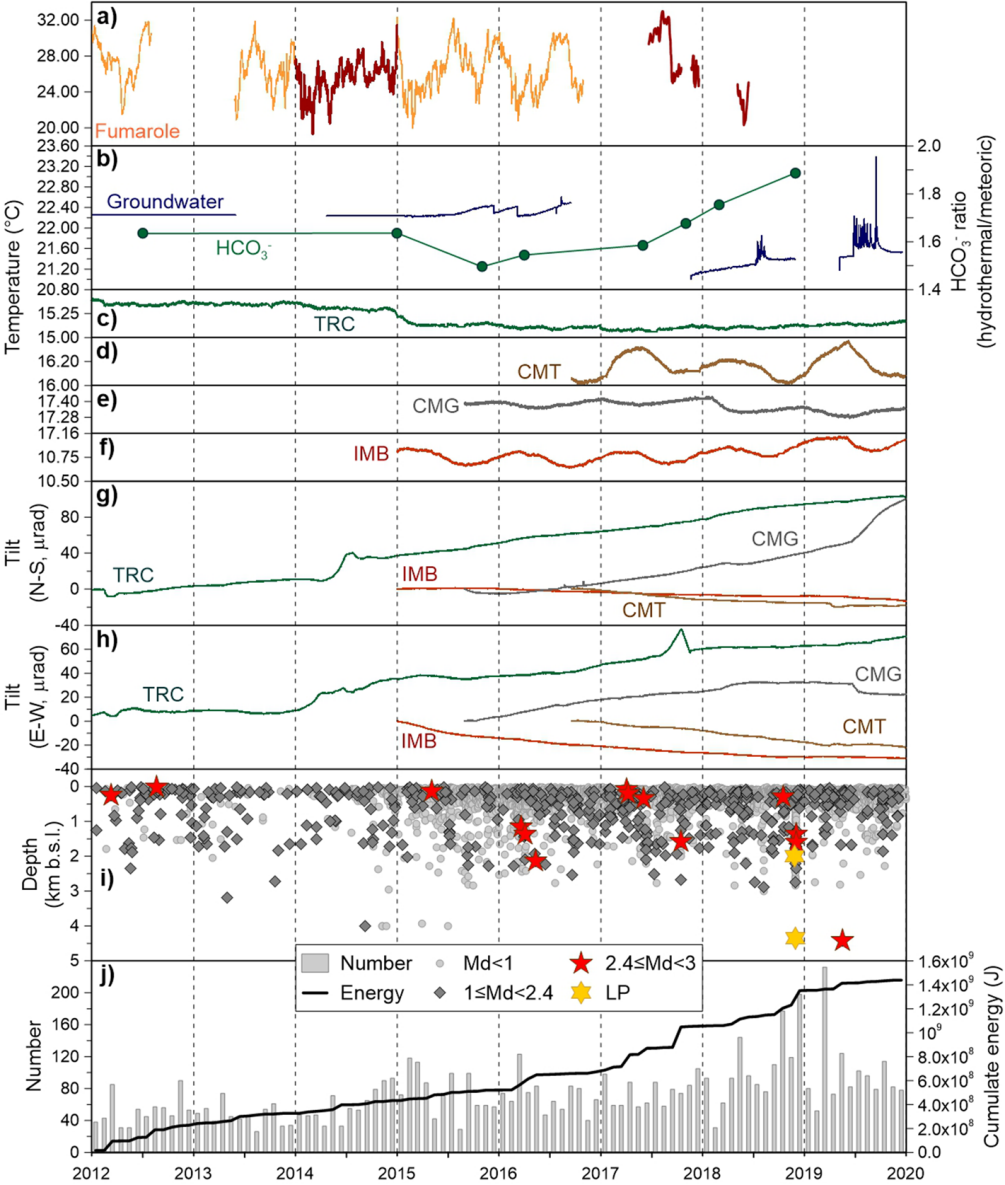
(**a**) Temperature of Gran Cono fumarole (dark red anomalous periods described in the main text, missing data due to sensor breakdown); (**b**) groundwater temperature (continuous data, missing values due to sensor breakdown) and bicarbonate content ratio between meteoric- and hydrothermal-dominant sites; (**c**)–(**f**) temperature recorded at tiltmeters TRC, CMT, CMG and IMB; (**g**) NS and (**h**) EW tilt components; (**i**) depth and binned magnitude and (**j**) number and cumulative energy of earthquakes.

We calculated the monthly number of VT earthquakes and energy using the seismic catalogue compiled for station BKE (Fig. 1), a long-established (1992-present) station with the best signal-to-noise ratio in the seismic network. The seismic energy was estimated by using the Gutenberg-Richter relationship^30^:

$$ {\text{Log}}\left( E \right) = {2}.{9 } + { 1}.{\text{9Md}} $$

where *E* is the energy in joules and Md is the duration magnitude. In Fig. 2j the temporal distribution of the number of VT events and energy is shown, accompanied by the temporal distribution of the VT hypocenter depths (Fig. 2i), obtained from the routine 1D location database of Mt. Vesuvius (www.ov.ingv.it).

During 2012–2014, the seismicity rate was low, as well as the energy release; the hypocenter depths were mainly in the upper 2 km until the end of 2014 (Fig. 2i).

In January 2015 there was a slight increase in the monthly number of earthquakes; on May 5th a shallow (< 1 km bsl) earthquake with Md = 2.5 occurred. Moreover (especially in the second part of the year), deeper hypocentral locations, in the 2–3 km bsl range, appeared; this continued until 2019 (Fig. 2i).

The year 2016 was characterized by the occurrence of three Md = 2.4 earthquakes at intermediate depths (1–2.5 km bsl) and relatively close in time (on March 3rd, April 4th and May 12th, respectively).

In 2017 there were two peaks in the energy release: the first, in April, is related to two extremely shallow (< 0.3 km bsl) earthquakes, both of Md = 2.4 (on April 4th and April 10th). The second, in October, is the highest energy peak of the investigated time interval (2012–2019) and is associated with a seismic swarm (on October 15th) composed of 13 earthquakes, with maximum Md = 2.8 and hypocentral depth of 1.6 km bsl.

**Figure 3:**
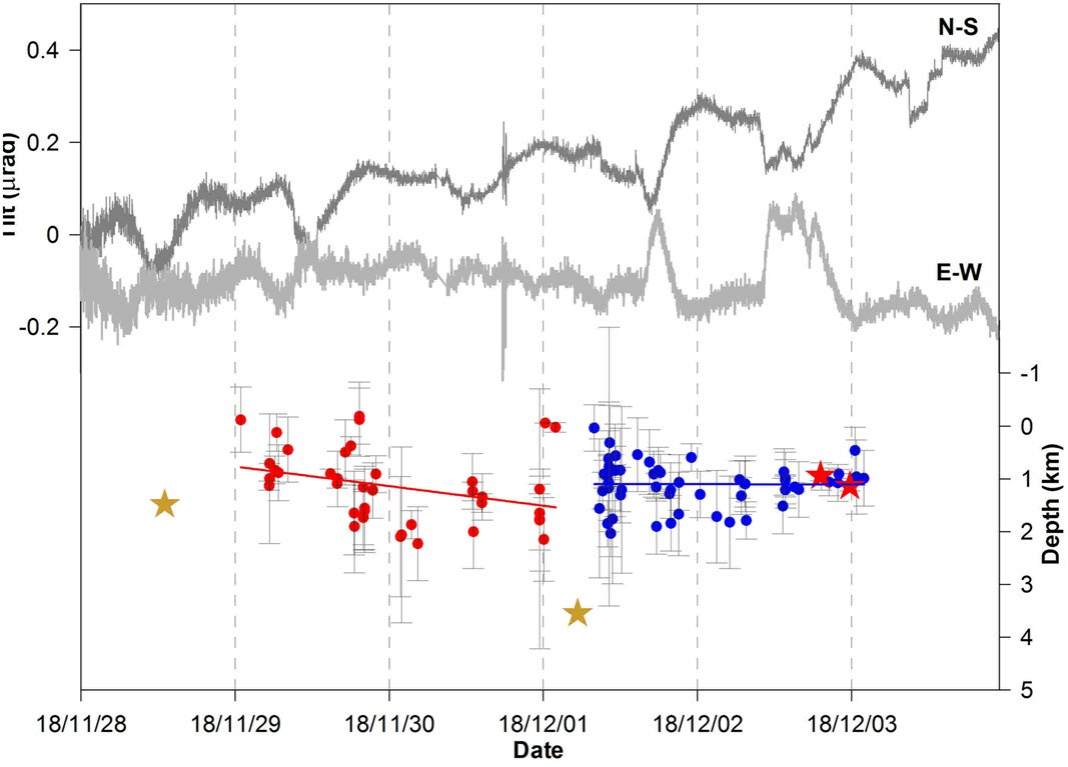
Lower curves show the temporal pattern of hypocentral depths of the November 29th-December 2nd 2018 seismic sequence. During the first two days, the VT depths (red circles) show a downward trend (red line), while during the last two days the VTs (blue circles) stabilized around 1.5 km of depth (blue line). The gold stars indicate the two LPs and the red ones mark the two Md = 2.5 VTs. The upper continuous lines represent the north–south and east–west components of ground tilt at CMG. Transients occurred on December 1st and 2nd, lasting 5 and 12 h, respectively. These tilt variations correspond to a ground inclination towards SE.

The year 2018, especially the second half, was marked by a general increase of both earthquake rate and energy. On October 16th a small swarm of 13 shallow earthquakes (< 1 km bsl) with -0.7 ≤ Md ≤ 2.5 was detected. On November 29th a seismic sequence consisting of about 120 VTs began. This sequence climaxed on December 2nd, with two Md = 2.5 earthquakes at a hypocentral depth of about 1.5 km bsl. The seismic events of the sequence were located beneath the crater area at depths between 0.7 and 2.6 km bsl; throughout the first two days the hypocenters show slight downward migration (Fig. 3). Analysis of their focal mechanisms indicates dominant shear components (http://www.ov.ingv.it/ov/it/bollettini/275.html). In addition, six earthquakes with low frequency content occurred on November 28th and two on December 1st (Figs. 2i, 3); these events were classified as Long Period (LP) by Petrosino and Cusano^24^. It is important to note that the energy content of the November/December LPs was about one order higher compared to events of the same types recorded in the past^24^.

During 2019 the seismicity remained at moderately high levels. In March, the earthquake frequency reached its highest level (more than 240 events) since 2012. On March 25th a seismic swarm consisting of 146 events with magnitude range -1.6 ≤ Md ≤ 1.6 intersected the crater area at shallow depths (< 1 km bsl)*.* The peak of energy release for the year 2019 occurred in May and was related to a Md = 2.5 deep earthquake recorded on May 18th. This earthquake was located east of the crater in the proximity of Mt. Somma and, with a hypocentral depth of 4.4 km bsl, it was the deepest VT since 2012.

**Figure 4:**
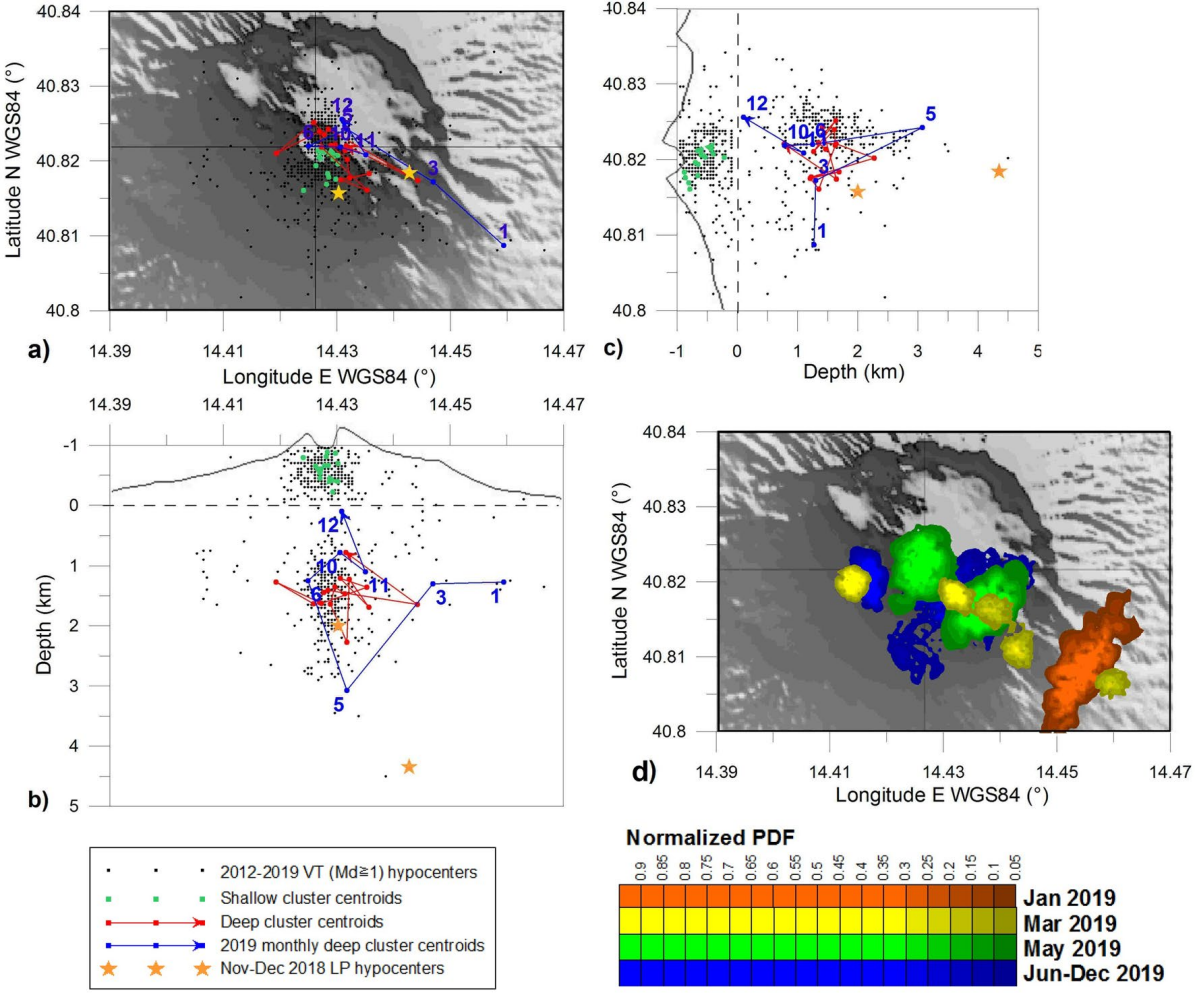
3D probabilistic location of 619 high energy (Md ≥ 1) VTs (387 in the shallow cluster and 232 in the deep one): (**a**) horizontal plane, (**b**) EW section, (**c**) NS section. Arrowheads indicate the point towards which the cluster is evolving. The blue labels of the hypocenter centroids specify the month of occurrence (1–12); missing labels indicate months with no earthquakes with Md ≥ 1. The legend is at bottom left. (**d**) Cumulative probability density function (PDF) for the VTs in 2019; different colors are associated with the months indicated in the legend at the bottom right.

We performed a 3D probabilistic location^31^ of the high energy (Md ≥ 1) VT earthquakes in 2012–2019. Because this process considers the volcano topography and lateral heterogeneities of the medium, the estimated locations allow us to track the time evolution of the hypocenters with a higher level of detail than the routine products. The calculated locations are represented in Fig. 4 by black dots (errors on the spatial coordinates are on the order of 0.3 km). As has already been discussed^19,24^, the hypocenters define shallow and deep clusters. To track the locations over time, we estimated the hypocenter centroid over two semi-annual periods (January-June and July-December) of each year from 2012 to 2019 for the shallow and deep clusters. It can be seen from Fig. 4 that the upper cluster is confined to a defined volume, while the lower one shows a more variable pattern. The point for the first six months of 2019 moves towards the SE sector of the volcano, far from the centroid cloud.

To further investigate this anomaly, we estimated the monthly centroid and the cumulative probability density function (PDF) for the year 2019. The hypocenter centroids for the deep cluster, shown in Fig. 4, are labelled according to the month of occurrence. The deviation began in January, with a centroid far from the crater axis (about 3 km) towards the SE direction. The anomaly persisted in March, with the centroid somewhat closer to the crater axis. In May the centroid returned to the deeper part of the cloud. The PDF distributions calculated for each of these months (Fig. 4d) indicate that the shift of the VT locations is significant. The last 6 months of 2019 show upward migration of the centroids. We performed the same analysis for 2018, but there were no unusual patterns.

In Fig. 4 we also plot the positions of the two locatable earthquakes belonging to the LP sequence at the end of 2018, obtained by Petrosino and Cusano^24^. By applying a polarization analysis combined with the same location procedure adopted in the present work, these authors found that the two LPs (on November 28th and December 1st) were located in the SE sector of the volcano (gold stars in Fig. 4).

### Ground deformation

The tilt time series provide the orientations of the plane tangent to the geoid at any measurement point (tiltmeter station locations). Rotations are related to micro-movements recorded at that site. If these orientations remain constant during a sufficiently long time, and instrumental (drift) or site problems (e.g. slow-motion landslides) can be excluded, they can be considered well-defined tilting directions.

**Figure 5:**
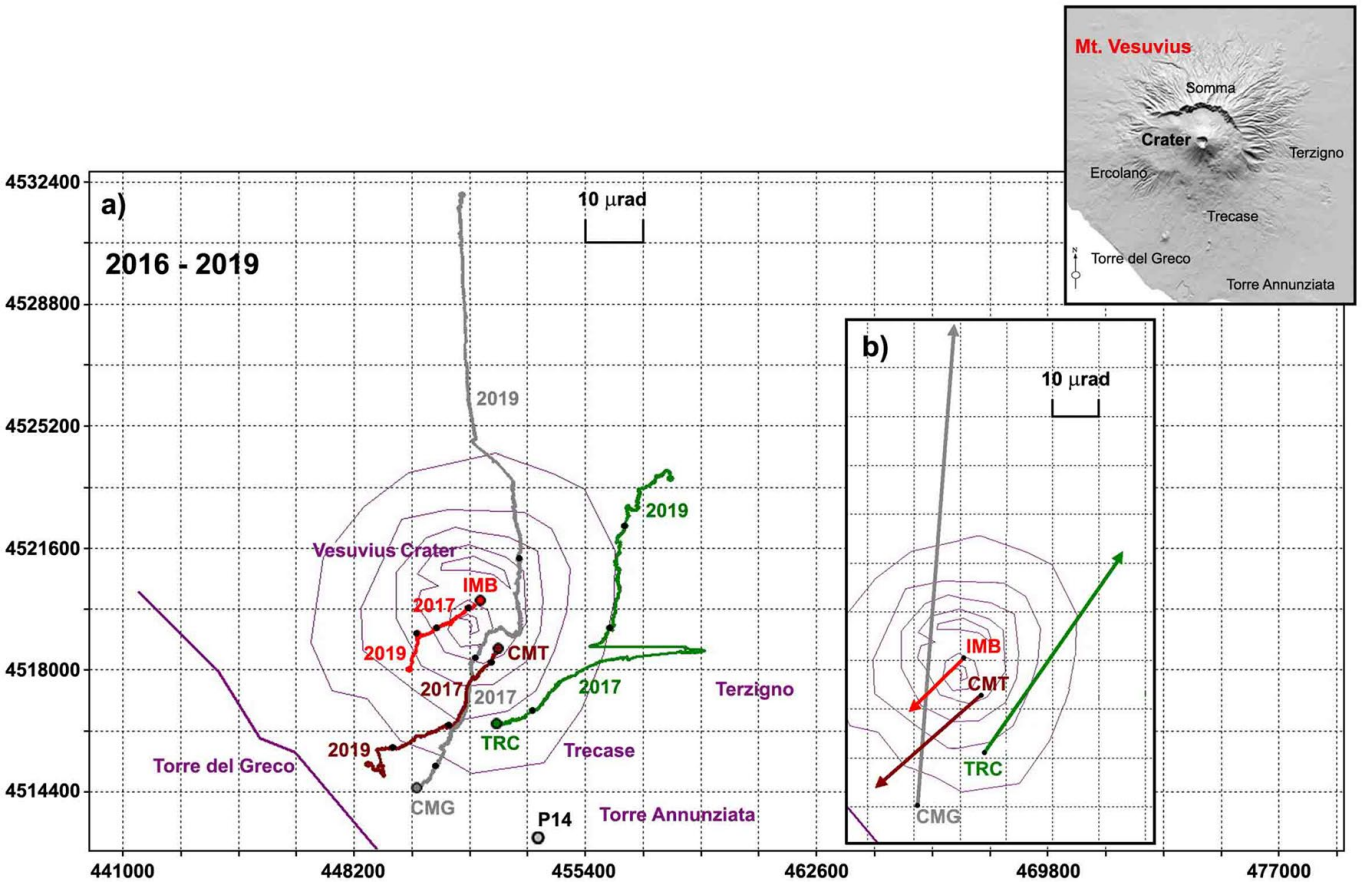
(**a**) Plots of tilt vectors at the four tiltmeters IMB (red), CMT (brown), TRC (green) and CMG (grey). The curves that originate from each station indicate the cumulative tilt variation (hodograph) recorded from September 18th 2016 to December 31st 2019. The black dots superimposed on the hodographs mark the recorded yearly tilt. The tilt increase on the NS component must be interpreted as northward down with respect to the site, the tilt increase on the EW component as eastward down. (**b**) The inset shows the dominant direction of ground tilting inferred from the hodograph.

Since 2012, the preferential tilt directions reflect a subsidence pattern affecting the Gran Cono of Mt. Vesuvius and its base, broadly consistent with that inferred from DInSAR (http://webgis.irea.cnr.it/) and GPS data (see the monitoring reports available at the URL http://www.ov.ingv.it/ov/it/bollettini/275.html). However, these main orientations were periodically interrupted by gradual or abrupt changes (Fig. 5).

From 2012 to 2016, the only borehole station operating at Mt. Vesuvius was TRC, and its main tilting direction was to the NNE. In 2014 this instrument recorded three changes of tilt orientation: on January 1st, April 5th and July 27th (Fig. 2g,h). In particular, on April 5th 2014 the TRC tilt changed from its usual NNE direction, bending towards the crater, along a trajectory oriented towards the Gran Cono. This tilting abruptly stopped on July 27th and gradually returned to its NNE direction (Fig. 2c–g). In the same year, the temperature sensor of the TRC station recorded a decrease of about 0.2 °C, which started on December 24th and ended at the beginning of March 2015 (Fig. 2c).

Since 2016 all four stations have been in operation simultaneously. The observed prevailing tilting directions from September 18th 2016 to December 31st 2019 were NNE at the two tiltmeters located in the southern area at low (CMG) and mid (TRC) elevation and SW at the two tiltmeters located at higher elevations in the northern area, north (IMB) and east (CMT) of the crater (Fig. 5).

**Figure 6:**
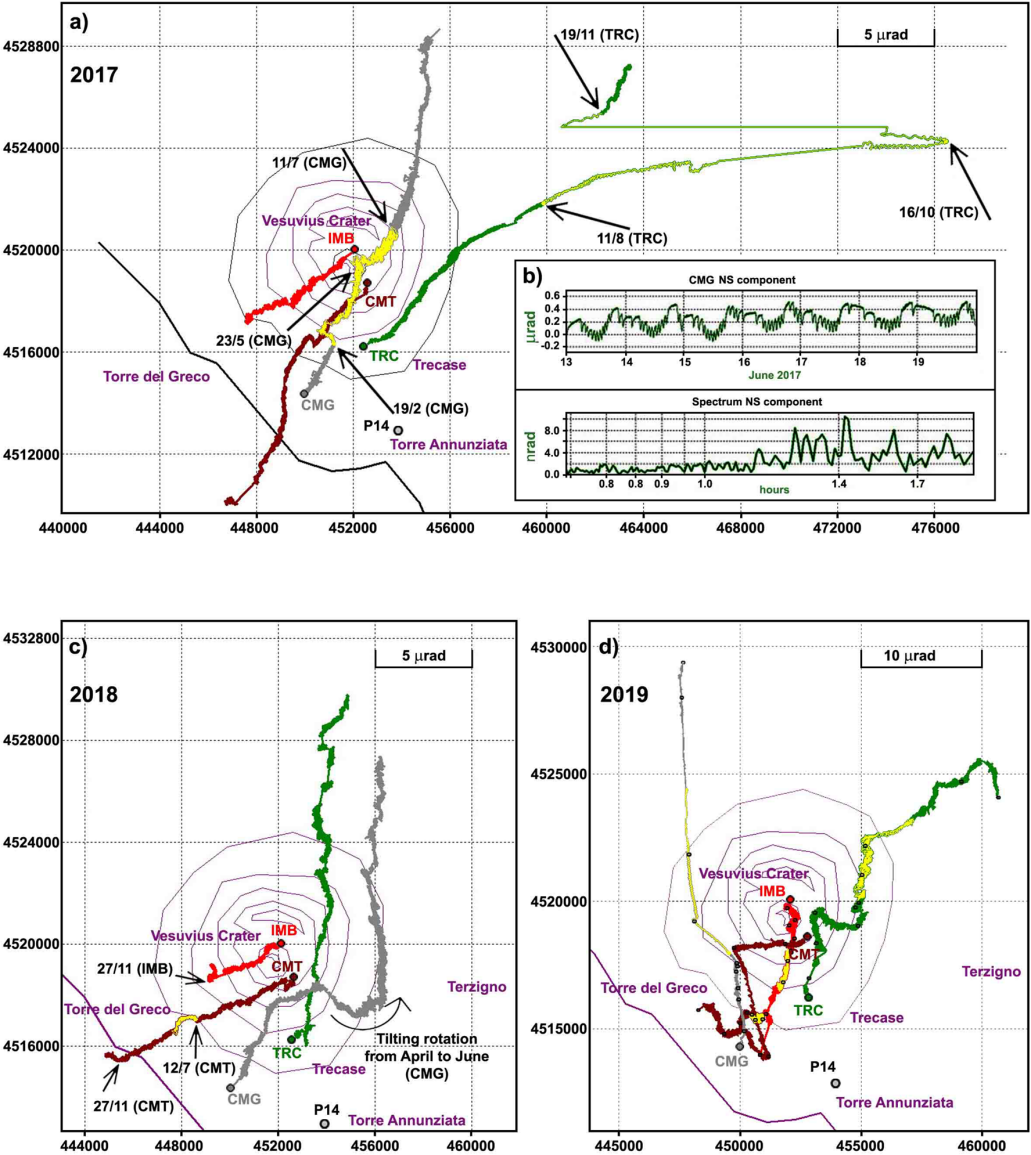
(**a**) Hodographs recorded at the four tiltmeters (cumulative variations) in 2017, with anomalous periods labeled with start/stop dates and highlighted in yellow; (**b**) signal sample and its spectrum recorded at CMG in June 2017, when the oscillations that appeared on May 23rd reached the maximum amplitude; (**c**) hodograph recorded in 2018 (anomalous period in yellow, arrows showing changes of tilt directions); (**d**) hodograph of 2019; yellow marks the anomalies recorded between June 25th and September 15th (CMG plot reduced by a factor 4 to facilitate comparison with other stations).

In 2017 these directions changed, for a limited time, at the two low-elevation tiltmeters (CMG and TRC) (Fig. 6a). At least three different anomalous events affected these stations, changing both tilt orientation and in one case also the spectral content of the signals (Fig. 6b). The first two events were observed at CMG (Fig. 6a, tilt in yellow): (1) a slow counterclockwise rotation of the tilting direction, from NNE to NW, occurred from February 19th through the first half of March and (2) non-tidal frequency oscillations in the two components (much more pronounced on the NS component) occurred from May 23rd until July 11th and reappeared intermittently in August. The oscillation period varied in the range 0.5–2 h, but the maximum spectral amplitudes fall in the band 1.2–1.4 h and occur during daylight hours (Fig. 6b). The hypothesis that a seasonal phenomenon, due to natural and/or anthropogenic causes, could have affected the time series was ruled out by checking past years’ records for the same period. The third event took place at the end of July, but became more prominent after August 11th (Fig. 6a, tilt in yellow). This event affected the TRC site, with a gradual change of the dominant tilting direction from NNE to E. This singular trend continued through the first half of October, but after October 16th, the day after the most energetic (Md = 2.8) seismic event of 2017, the eastward drift ended, and the tilting direction rotated counterclockwise, gradually recovering part of the accumulated deformation since August 11th. A few days later a modest landslide hit an area about 140 m west of the TRC site, taking the EW component of the tiltmeter off scale. After recalibrating the sensor, the ground inclination gradually returned to the NE direction (Fig. 6a).

During early 2018, the inclination of the volcanic edifice followed the established pattern. The first noteworthy tilt change occurred at CMG in April, when the tilting direction deviated from the dominant N–NE direction by turning 90° clockwise (Fig. 6c)_**.**_ From mid-June, it started tilting northwards again (semicircular arrow in Fig. 6c). No particular anomalies occurred later, except for a large oscillation in the direction of the crater recorded at CMT, which started on July 12th and lasted about 40 days (Fig. 6c, tilt in yellow). After November 27th there was a change in tilting direction that affected the higher stations: the NS component of IMB reversed its tilting direction pointing to the N, and the same phenomenon, less marked, affected the homologous CMT component (arrows in Fig. 6c).

**Figure 7:**
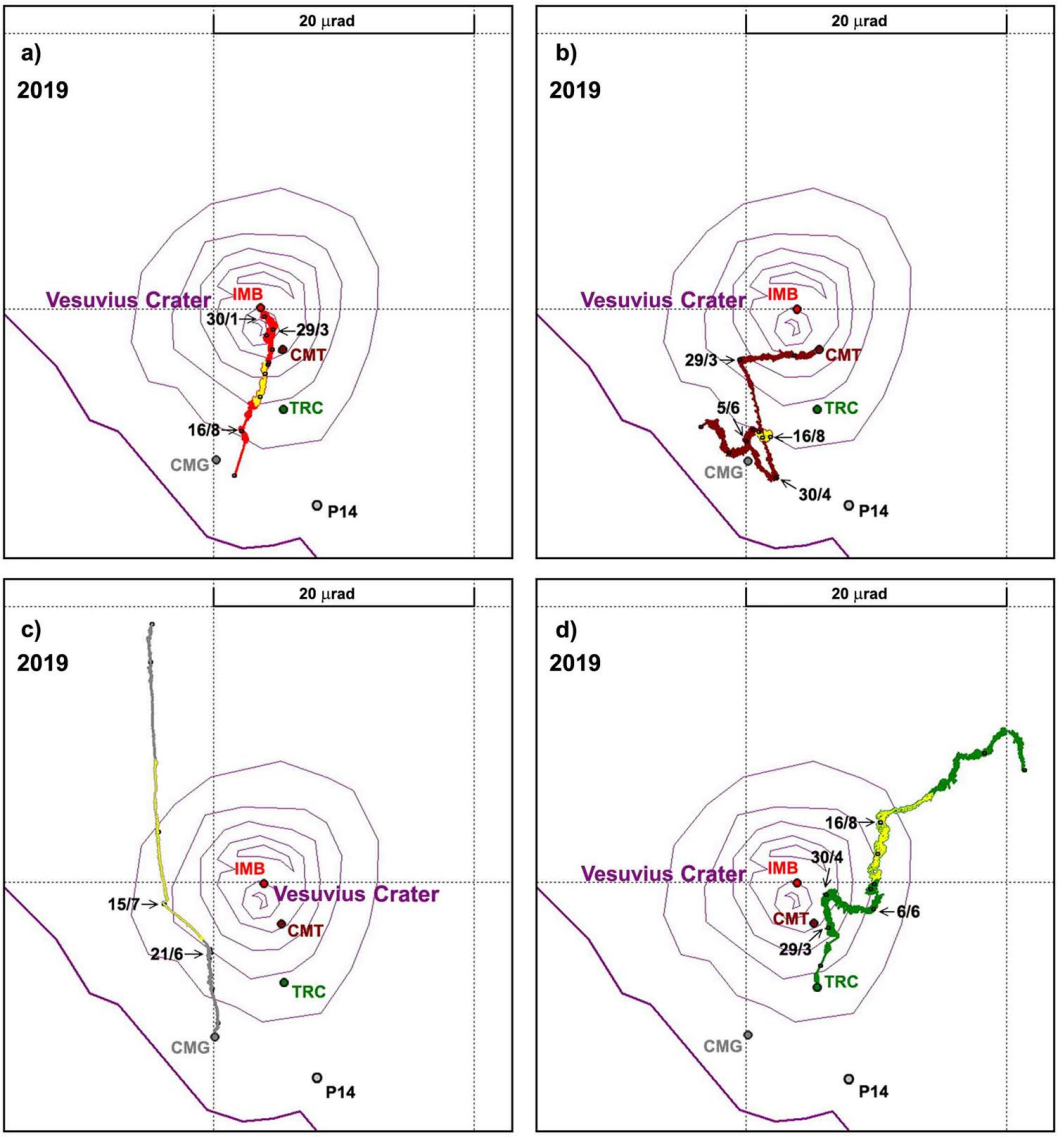
The 2019 hodograph emphasizing the time-evolution of ground tilt at (**a**) IMB, (**b**) CMT, (**c**) CMG and (**d**) TRC. The dates indicate the most important changes of direction and amplitude recognized on the cumulative tilts. The yellow color marks the anomalies recorded between June 25th and September 15th.

**Figure 8:**
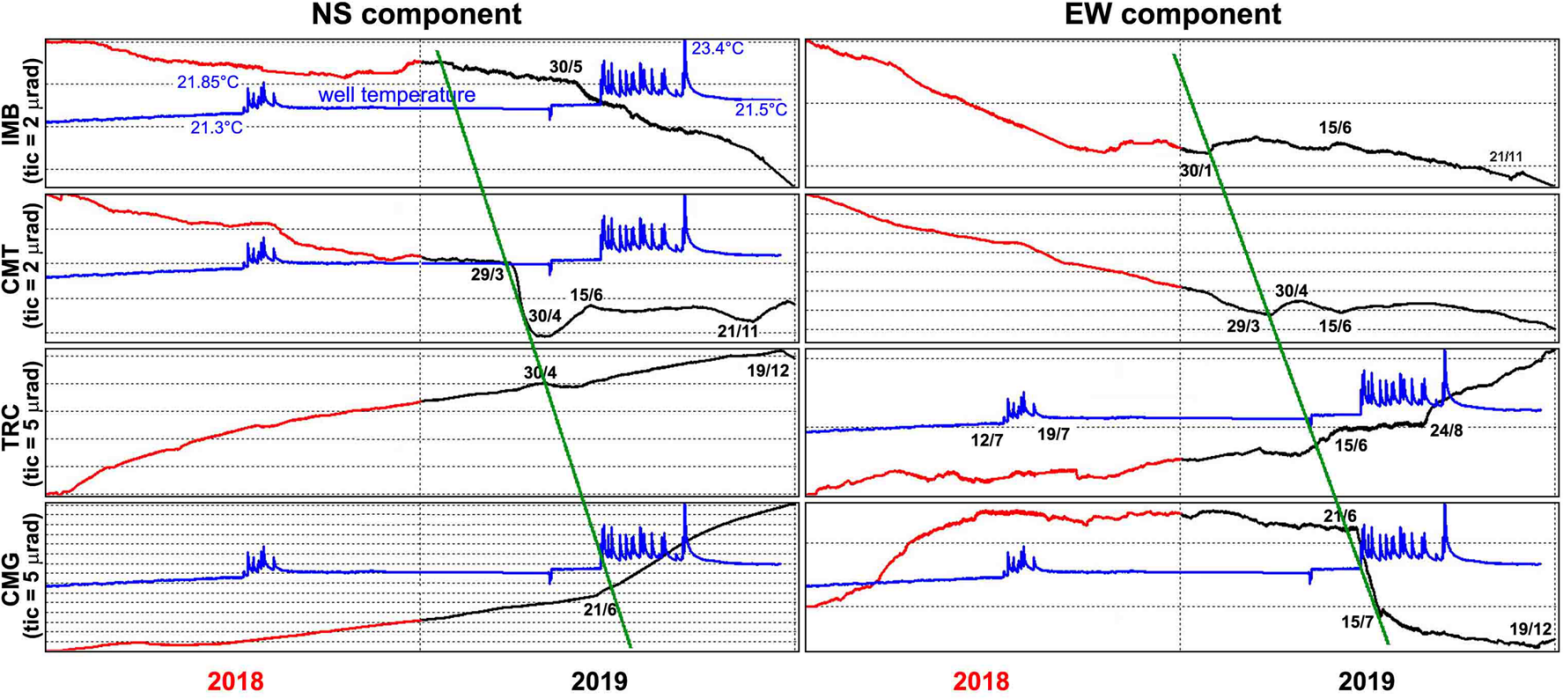
Tilt signals for the years 2018–2019 sorted, from top to the bottom, by decreasing elevation of tiltmeters. The groundwater temperature recorded at well P14 is reported in blue for comparison. To highlight the time sequence of the anomalies, two green lines with the same slope have been superimposed on the NS and EW signals and intercept evident variations of the ground tilt on the NS and/or on the EW components.

During 2019 the tilt pattern (Fig. 6d) was characterized by greater complexity compared to previous years. This behavior affected all stations at different times but sometimes also simultaneously. We recorded at least five tilt anomalies: January 30th (EW component of IMB), March 29th (both at CMT and in the EW component of TRC), April 30th (both at CMT and TRC), June 21st (CMG) and July 15th (EW component of CMG) (Figs. 7a–d, 8). The first of these anomalies was a tilt rotation at IMB (Figs. 6d, 7a). The second relevant tilt change was observed at CMT beginning in March 29th, and consisted of a counterclockwise tilting reversal which ended on April 30th, with a further reversal on hourly scale (Figs. 6d, 7b). On that date a similar but symmetrical event (third anomaly) was observed at TRC (Figs. 6d, 7d); both phenomena ended on June 15th (Figs. 6d, 7b,d). Finally, on June 21st, the CMG station showed a decisive counterclockwise rotation (fourth anomaly) of its tilting direction, which was progressively recovered with a further clockwise rotation (fifth anomaly) on July 15th (Figs. 6d, 7c). The exceptionally large tilt rates observed at CMG since June (which forced us to rescale its hodograph in Figs. 6d, 7c), indicates kinematics affected by some other process besides the subsidence pattern of the Gran Cono and its base.

In order to understand whether a relationship exists among the tilt anomalies recorded at the different tiltmeters, we aligned the homologous components according to the elevation of the respective sites (Fig. 8). This arrangement of the time series makes it possible to see how the anomalies progressively affected stations at lower elevation, indicating deformation propagating from the Gran Cono towards its base (green timelines in Fig. 8) in the S-SE direction, with an estimated apparent velocity on the order of 40 m/day.

### Geofluid circulation

Data related to fumarole and groundwater temperature and chemistry (Fig. 2a,b) exhibit anomalies well correlated with those described for seismic and tilt signals. The first was observed in 2014 and concerned fumarole temperatures (Fig. 2a). The peculiar characteristic of this signal is a 6-month cycle, with two relative maxima in December-January and July–August. The summer maximum is related to the higher ground surface temperatures, controlled by the yearly sun irradiance cycle. The winter maximum is seemingly related to concentration of vapor flux through fractures: due to the high soil moisture content occluding the pores of the volcanic deposits, outgassing through the primary porosity is strongly limited during the winter. A similar winter maximum was observed by Caliro et al*.*^28^ for the CO_2_/H_2_O ratio in the Mt. Vesuvius fumaroles. The authors attributed this phenomenon to the condensation of water during the cold season, while our data suggest a different interpretation: the fumarolic fluid flux as a whole is concentrated along fractures, but the effect of water condensation due to lower ambient (and soil) temperatures causes the relative enrichment in CO_2_. In 2014 (evidenced in dark red in the Fig. 2a) there was no summer maximum, and the tiltmeter TRC (the only one operating at that time) showed significant variations of both the NS and EW components (Fig. 2g,h), followed by diminution of the temperature recorded in the instrument case between the end of 2014 and the beginning of 2015. These anomalies coincided with an almost total lack of the deeper seismicity (Fig. 2i).

The year 2015 was characterized by other anomalies related to geofluid circulation. Groundwater temperature continuously monitored in well P14 (see Fig. 1), which had remained very constant during the previous years, started to get warmer in mid 2015 (Fig. 2b), first with two consecutive “donkey-back” (slow increases followed by faster decreases) cycles and later, from early 2016, following a continuous positive trend. Moreover, the ratio between bicarbonate concentration in groundwater receiving a higher influx of the hydrothermal component (SW sector, see Fig. 1) and bicarbonate concentration in dominantly meteoric groundwater (NE sector) reached a minimum at the end of 2015 (Fig. 2b), due to contemporary concentration increases in the SW sector and decreases in the NE.

The second anomalous period with respect to geofluid circulation, began in mid 2017 and lasted until the second half of 2019. Unfortunately, the exact onset cannot be uniquely identified, due to the lack of continuous data caused by sensor breakdowns. The first evidence was an abrupt decrease of fumarole temperatures in the first days of September 2017 and lasting until the end of the available data (dark red curve of Fig. 2a). The years 2017–2018 were characterized by the highest air temperatures ever recorded, so that the trend shown by fumarole data was in counter tendency with respect to the atmospheric signal. The fumarole thermal anomaly was paralleled by a contemporary and constant increase of the bicarbonate ratio in groundwater (Fig. 2b).

The behavior of groundwater temperature was more articulated (Fig. 2b). When data were available again (mid 2017), temperatures were again following a constant rate of increase starting from an initial value of 21 °C, about 1.3 °C lower than the previous minima. This constant trend was modulated by two clusters of short-lasting, positive pulses up to 23 °C, with frequency and amplitude never observed before. The first occurred in mid 2018, following a negative variation of temperature (Fig. 2e) and change of tilt (both components, Fig. 2e,g,h) at CMG. The second cluster occurred in mid 2019 and, like the previous one, was accompanied by concurrent anomalies in the tiltmeter network (Figs. 6d, 7). At its onset, CMT (Fig. 2d) and IMB (Fig. 2f) recorded their maximum observed temperatures. Moreover, tilt variations were recorded immediately before and at the onset of the temperature pulse cluster at CMT and CMG, respectively (Fig. 8).

## Discussion

Seismic, tiltmeter and geochemical data acquired at Mt. Vesuvius over the past eight years reflect the dynamics of its magmatic/hydrothermal system, characterized by both trends at medium time-scale (years) and short-lasting (months) anomalies.

The first notable event appeared in the first half of 2014, and consisted of concurrent and coherent changes in the behavior of all the acquired signals, indicating diminution of the summit hydrothermal activity. Between April and July, tilt variations indicate subsidence in the area between TRC and the Gran Cono crater (Figs. 1, 2g,h). Fumarole temperatures did not exhibit their seasonal summer maximum, normally occurring in July–August (Fig. 2a); seismicity was extremely shallow and not energetic (Fig. 2i,j). At the end of the year the temperature at TRC exhibited a sharp decrease (Fig. 2c), followed in 2015 by a diminution of the bicarbonate content in wells with a more hydrothermal signature (Fig. 2b). These signals suggest that the hydrothermal system was transferring fluids towards the surface at a slower rate. This evidence would be consistent with the general decreasing trend of Mt. Vesuvius activity, observed after the 3.6 tectonic earthquake of October 9th 1999^18,22,28^.

Indications of a new phase of increasing activity began to be recorded in 2015. After more than 3 years of stability, the groundwater temperature in well P14 (Figs. 1, 2b) started to be modulated by cyclic increments, followed by sudden decreases (Fig. 2b). In the same period (since early 2015), seismic activity (especially in the deeper cluster) started to increase, although a more substantial rise of seismic energy was observed in February 2016, as evidenced by the slope change of the cumulative energy curve (Fig. 2j). In contrast, the bicarbonate content continued its decreasing trend until the second half of 2016 (Fig. 2b).

The new phase of renewed volcanic activity has continued. Since February 2017, the southeastern sector of Mt. Vesuvius first exhibited uplift and later ground oscillations, a peculiar phenomenon that in recent studies has been related to fluid involvement in the dynamical processes of hydrothermal/volcanic systems^3,32,33^ (Fig. 6a,b). In August-October, a marked eastward rotation occurred in the ground tilt direction in the southeastern area, followed by a landslide. These tilt variations were accompanied by larger earthquakes (two Md = 2.4 shallow VTs in April, and one Md = 2.8 in October, Fig. 2i) and fumarole temperature changes (Fig. 2a). Moreover, the carbonate content curve changed from a smooth positive slope, maintained since its minimum of late 2015, to a steeper rate of increase (Fig. 2b) following the October 2017 earthquake; this trend continued until the last available data (end of 2018).

In 2018, concurrent tilt, seismic and geochemical anomalies continued to occur. From April to June the tilt at the southern foot of the volcanic edifice underwent a marked eastward rotation; then in July a large oscillatory tilt towards the crater occurred at the CMT site (Fig. 6c). In the same period, large-amplitude and short-duration pulses started to affect the temperature signal at P14 (Fig. 2b). In November–December high energy LPs, located southeastward of the crater (Fig. 4), preceded and accompanied energetic VTs, which occurred mainly in a seismic sequence along the crater axis at increasing depths (Figs. 2i, 3). The CMG tiltmeter recorded a co-seismic ground tilt towards SE (Fig. 3) during this time. Moreover, beginning in November the summit area was affected by tilt reversals (Fig. 6c).

All these variations clearly depict a renewed dynamics of the hydrothermal system of Mt. Vesuvius, also highlighting that structural changes were taking place. Thermal pulses in groundwater suggest injection of hot fluids into the basal carbonate aquifer; at the same time the negative trend (red curve in Fig. 2a) in temperature at the summit fumarolic field indicates a decrease in vapor flow from the second half of 2017 to the first half of 2018 (end of available data due to sensor breakdown). These behaviors can be reconciled if we hypothesize that the centroid of the surface manifestation of hydrothermal activity began to migrate southeastward of the crater area. This hypothesis is supported by the ground tilt pattern at the CMG site, at low elevation on the southern foothills of the volcano, which suddenly changed its orientation towards well P14 (Fig. 6c) about 2 months before the first cluster of temperature pulses (Fig. 2b). Stress changes related to fluid circulation could have driven the LP activity in the southeastern sector of the volcano, as well as the downward migration of the hypocenters of the VT sequence. It is likely that, as observed in other volcanoes, the direction of earthquake migration reflects the pattern of the fluid circulation^4,6,16^. Assuming that the seismic sequence of November/December 2018 can be modelled as a process of pore pressure diffusion^34^, we calculated the hydraulic diffusivity, *D*, by fitting the rate of deepening of the hypocenters to the equation:

$$ r = 4\pi Dt $$

where *r* is the distance at which the triggering pressure front has arrived at time *t*. Due to the strong clustering along the crater axis, *r* can be substituted by the hypocentral depth. The obtained diffusivity value is in the range 1.9–2.9 m^2^/s, at a confidence level of 95%; this parameter is related to the medium permeability, *k*, according to^35^:

$$ k = D\eta \beta \phi $$

where η, β are the fluid viscosity and compressibility, respectively, and ϕ is the rock porosity. By using the same values assumed by Ventura and Vilardo^36^ for Mt. Vesuvius, we calculated a permeability range of 4–6 × 10^–13^ m^2^, within the range of measured geothermal-reservoir permeabilities worldwide^37^. Our estimates of diffusivity and permeability are higher than those obtained by Ventura and Vilardo^36^ (diffusivity 0.25 m^2^/s, permeability 5.3 × 10^–14^ m^2^) and Saccorotti et al*.*^34^ (diffusivity 0.18–0.28 m^2^/s), who calculated those parameters for seismic swarms occurring from 1995 to 1999. These results suggest that the seismogenic volume of the 2018 sequence was affected by a higher degree of fracturing, possibly induced by fluid pressurization^38,39^.

Additional indications of the southeastward migration of hydrothermal activity were recorded in 2019. In January strong subsidence of the crater area was detected, and in the first four months of 2019 this deformation pattern propagated towards lower elevations; the phenomenon was paralleled by southeastward migration of the earthquake centroid. Between the end of April and May these signals became even more intense: a supplementary rotational deformation at low elevation overlapped with the subsidence, in conjunction with a very deep high energy earthquake (on May 18th), still located east of the crater. Finally, by the end of June, the well P14 showed a new sequence of temperature pulses (Fig. 2b), similar to those observed in the summer of 2018.

**Figure 9:**
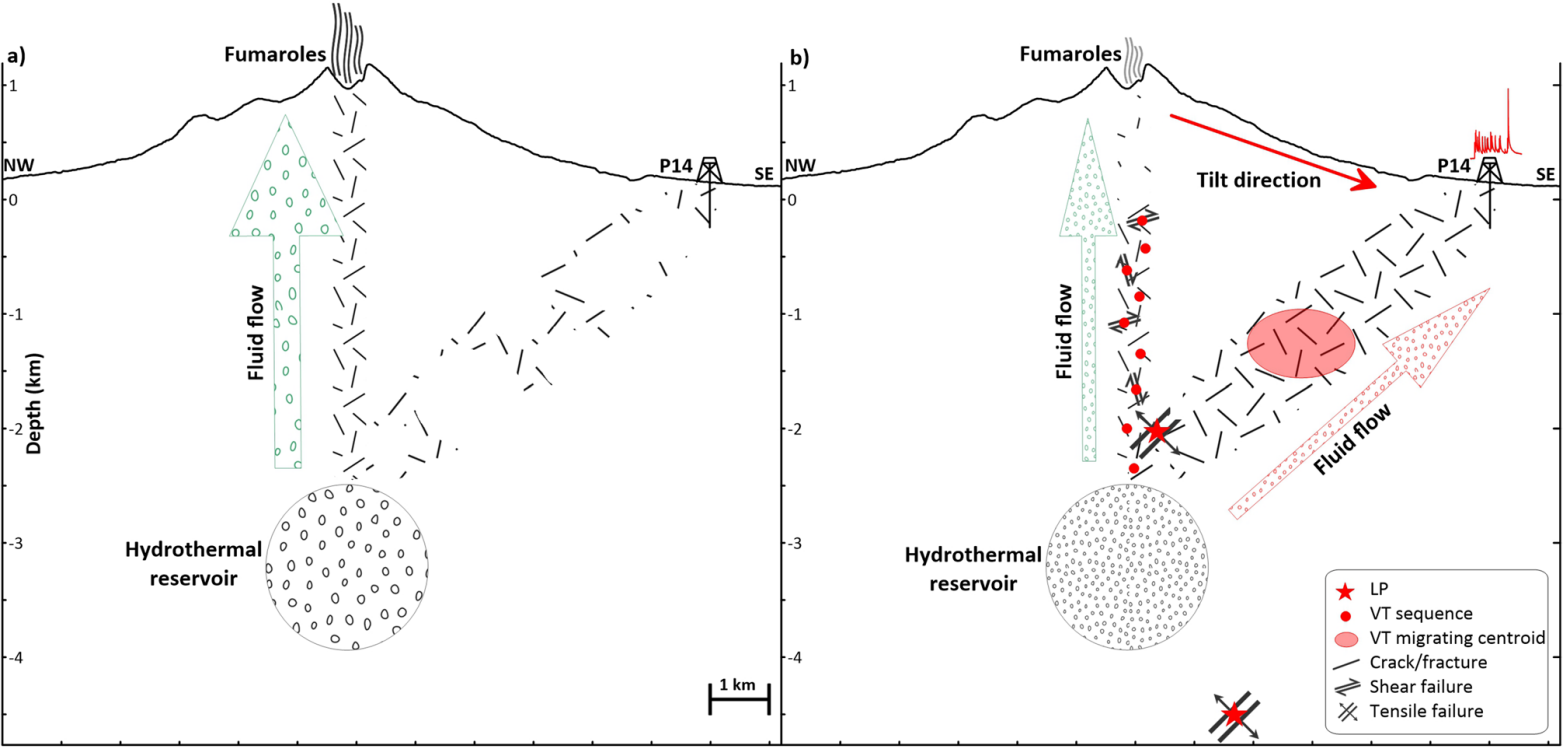
Conceptual model overlaid on the NW–SE section of Mt. Vesuvius: (**a**) in the background state, fluids sourced from the hydrothermal reservoir mainly propagate (green arrow) through the fractured volume beneath the crater axis, feeding the summit fumarolic field and (**b**) pressure variations in the hydrothermal system induce crustal strain that restricts some pathways towards the crater, decreasing the fumarole outgassing. Fluid pressurization causes VTs and LPs and opening of new flow pathways (red arrow) in a pre-existing zone of weakness. As a result, the manifestations of volcanic activity (deformation, seismicity and groundwater thermal anomalies) migrate SE.

The number and strength of the geophysical and geochemical indicators detected during this last phase*,* beginning in October/November 2018, lead us to hypothesize a major change in the migration paths of hydrothermal fluids, from the Gran Cono crater area towards the base of its southeastern outer flank. This phase can be described by a conceptual model (Fig. 9) according to which the observed phenomena are the effect of significant pressure variations in the hydrothermal reservoir. Such variations promote crustal strain changes (deformation and seismicity), which in turn affect fluid circulation^40,41^. Crustal strain and/or sealing due to hydrothermal alteration^39^ likely forced the closure of some pathways towards the crater area. As a consequence, fluids found new ways of escape. The fluid pressurization reactivated fractures by shear failure and cracks by tensile failure. The first mechanism triggered the VT sequence along the crater axis, enhancing the medium permeability (similarly to what happened during the swarms in 1994–1999) ^34^, whereas tensile failure of fluid-filled cracks generated the LP events^24^. The opening of new flow pathways was particularly efficient in the southeastern sector of the volcano, possibly due to pre-existing weakness. Fracture lubrication, crack expansion, and thermoelastic stress^42^ could have combined to channelize fluids towards SE, as evidenced by the ground deformation pattern, LP occurrence, anomalous location of VT centroids, and groundwater thermal anomalies in the peripheral well P14. Other mechanisms generating crustal strain, such as the gravitational slumping of the volcanic edifice and /or changes in the regional tectonic stress field, cannot be ruled out.

In many volcanic areas, variations in the activity of the hydrothermal system and of volcano dynamics have been detected through observations of geophysical signals^5,43–47^. Ground deformation, seismic activity variations, and heat flux changes do not necessarily involve magma migration towards the surface, leading to eruptive processes, but can be also driven by the transfer of fluids and heat from a magmatic body remaining at depth^4,45,48^. In this case, the geophysical signals are likely poroelastic and thermal responses of the medium to fluid-rock interactions^3,43,45,48^.

The role of hydrothermal fluids in past eruptions of Mt. Vesuvius has been pointed out by Bertagnini et al.^12^ in their study of the 1906 event. These authors suggest that repeated fluid blasts, caused by the flashing of the hydrothermal system, played a very important role, and occurred from lateral vents which opened south-eastward of the Gran Cono crater at an elevation of about 800 m asl, near the remnants of the ancient crater rim of Mt. Somma^49^.

The rock volume weakened by the 1906 eruption, located on the SE flank of the Gran Cono, may represent a preferential path for the circulation of hydrothermal fluids, promoted by both subsurface (cracks and fractures) and surface (crater rim remains) structural discontinuities. The presence of this peripheral, preferential hydrothermal circulation path could explain the observed southeastward migration of the centroid of the manifestations (seismicity, differential ground tilt response to strain variations, thermal and compositional anomalies in groundwater and fumaroles) of volcanic activity. These considerations lead to an important inference about the possible location of future eruptive centers. The 1906 eruption originated from vents located in an eccentric position with respect to the Gran Cono crater axis, where volcanic activity returned in 1944. The present SE migration of hydrothermal activity suggests that the 1906 eruptive centers’ area might be involved again in future eruptive events.

Thanks to joint investigations of the episodic variations in geophysical and geochemical signals, we were able to track, for the first time since the advent of modern technologies for the monitoring of Mt. Vesuvius, significant changes in the activity state of the volcano. Such interdisciplinary investigations represent a useful tool for volcano surveillance, opening promising new perspectives.

## Methods

In this section, the data used in the present study are briefly described. The data were collected by the monitoring system of the Istituto Nazionale di Geofisica e Vulcanologia (INGV). Several networks (Fig. 1) provides seismic, ground deformation and geochemical data.

### Seismic data

Mt. Vesuvius seismic network is currently composed of 19 permanent and 6 temporary standalone stations. The permanent dataloggers are three-component digital broadband or analog short-period devices (Fig. 1). The acquired data are telemetered in real time to the surveillance center of the INGV, Sezione di Napoli – Osservatorio Vesuviano (INGV-OV). The temporary stations are three-component digital broadband dataloggers that store the data locally. For details see^50,51^.

The permanent short-period BKE station is generally taken as a reference because it presents the best signal-to-noise ratio of the entire network. It is located on the eastern flank of Mt. Vesuvius, close to the crater, and is one of the first modern monitoring instrument installed on the volcano.

The seismological dataset consists of the VT earthquake signals recorded in the time range 2012–2019, the seismic catalogue containing the duration magnitude values for BKE station (http://www.ov.ingv.it/ov/it/banche-dati/186-catalogo-sismico-del-vesuvio.html) and the routine 1D locations of the VTs (http://www.ov.ing.it).

### Ground deformation data

Ground tilt monitoring on Mt. Vesuvius began in 1993, and until 2011 tilt variations were recorded by analog surface tiltmeters, the first of which was set up in the Vesuvius Observatory bunker. Later, in 1996, two other identical stations were emplaced near Torre del Greco (CMD) and near Trecase (TRC). Finally, at the end of 2011, the Vesuvius tilt network was extended by drilling four wells more than 20 m deep^26^. The first borehole instrument (TRC) replaced the preceding installation in November 2011; the other three borehole tiltmeters became operational in January 2015 (IMB) and September 2016 (CMG and CMT)^52^. The current network, managed by the INGV-OV, consists of seven instruments of two different types: three surface short base-length platform AGI 702 tiltmeters installed in shallow wells, and four borehole digital Lily tiltmeters. The latter have a cylindrical shape and are made of stainless steel; at the bottom, bubbles electrolyte, temperature sensor and the magnetic compass (to detect the change of magnetic declination) are positioned. The sensing device is a self-levelling sensor on a range of ± 10 degrees, with a dynamic range of ± 330 μradians and a resolution less than 5 nradians^53^.

The heights and installation depths of the borehole tiltmeters are: IMB (974 m asl, − 22 m ), CMT (842 m asl, − 20 m), TRC (372 m asl, − 28 m) and CMG (117 m asl, − 25 m).

### Groundwater and fumarole temperature data

Data of bicarbonate content and temperature of groundwater, and temperature of the summit fumarolic field, have been collected on behalf of the volcanic surveillance program of Mt. Vesuvius developed by INGV for the Italian National Civil Protection Department. Groundwater temperatures have been measured on an hourly basis using Gemini Tinytag Aquatic 2 loggers, with a resolution of 0.03 °C, inside an abandoned drilled well (P14, Fig. 1), thus not influenced by water extraction. Bicarbonate contents were determined in the INGV-Sezione di Palermo laboratory using an automatic titrator, in samples collected in the field and stored in hermetically capped 100 cc plastic bottles and kept refrigerated until the analysis, which is carried out within a few days from the collection. Sampling campaigns were conducted on a six-months basis, but we present only data related to sessions during which all the sites were sampled, to avoid pseudo-variations due to missing data.

Fumarole temperature were acquired on an hourly basis using Onset Hobo 4-channel dataloggers, equipped with 12-bit temperature smart sensors (resolution 0.03 °C).

## Data Availability

All relevant data are available from the authors.
